# Supplementary material for: Biosynthetic Nanobubble-Mediated CRISPR/Cas9 Gene Editing of Cdh2 Inhibits Breast Cancer Metastasis
Source: Pharmaceutics. 2022 Jun 30;14(7):1382. doi: 10.3390/pharmaceutics14071382 (PMC9319454; doi:10.3390/pharmaceutics14071382)
Supplement: Supplementary file 1 [file pharmaceutics-14-01382-s001.zip › pharmaceutics-1690686-SI.pdf]

pharmaceutics

MDPI

Supplementary materials: Biosynthetic nanobubble-mediated CRISPR/Cas9 gene editing of Cdh2 inhibits breast cancer metastasis

Ruru Gao [1,†], Qiong Luo [1,†], Yang Li [3,†], Liming Song [4], Junnan Stephen Cai [5], Ying Xiong [1], Fei Yan [2,*], and Jianhua Liu [1,*]

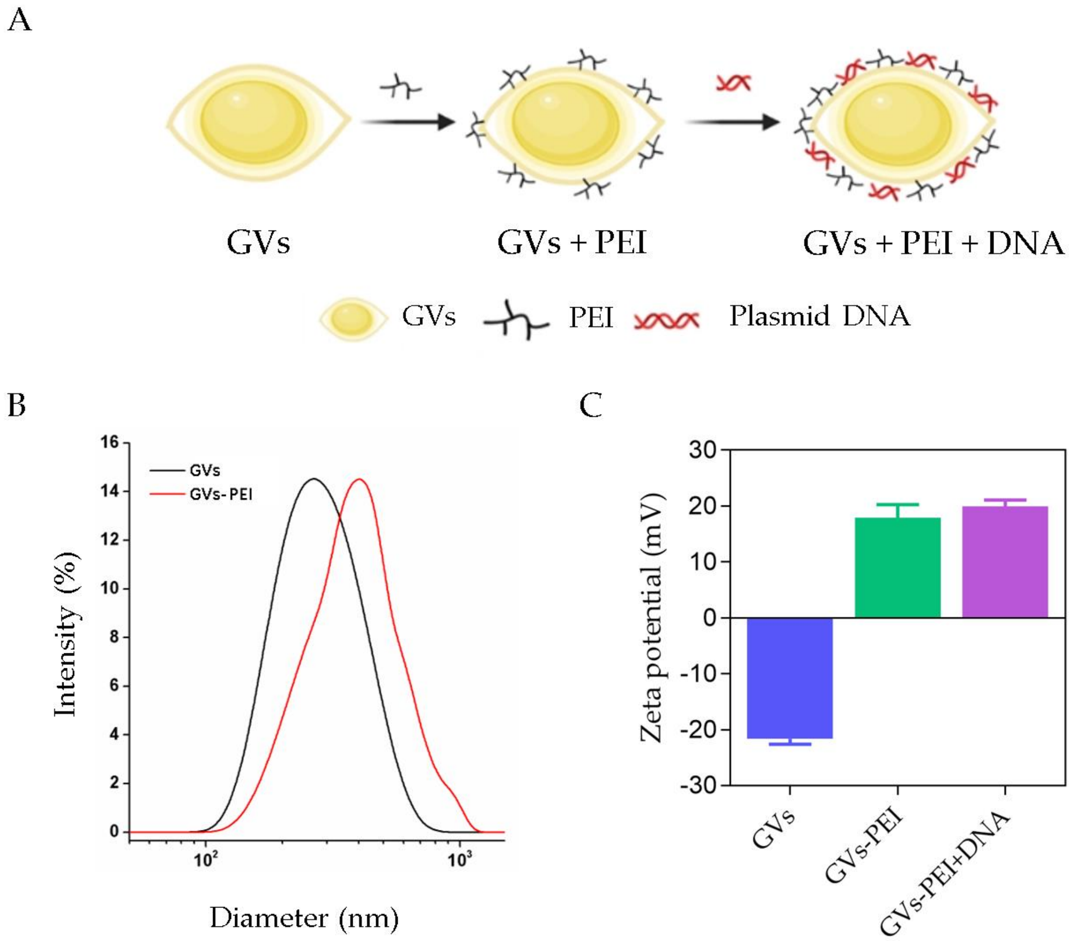

Figure S1. Preparation and characterization of GVs-PEI-DNA(GPD). (A) Diagram depicting the GPD's preparation process. (B) Size distribution of GVs and GVs–PEI. (n = 3). (C) Zeta potential of GVs, GVs-PEI, and GPD. (n = 3).

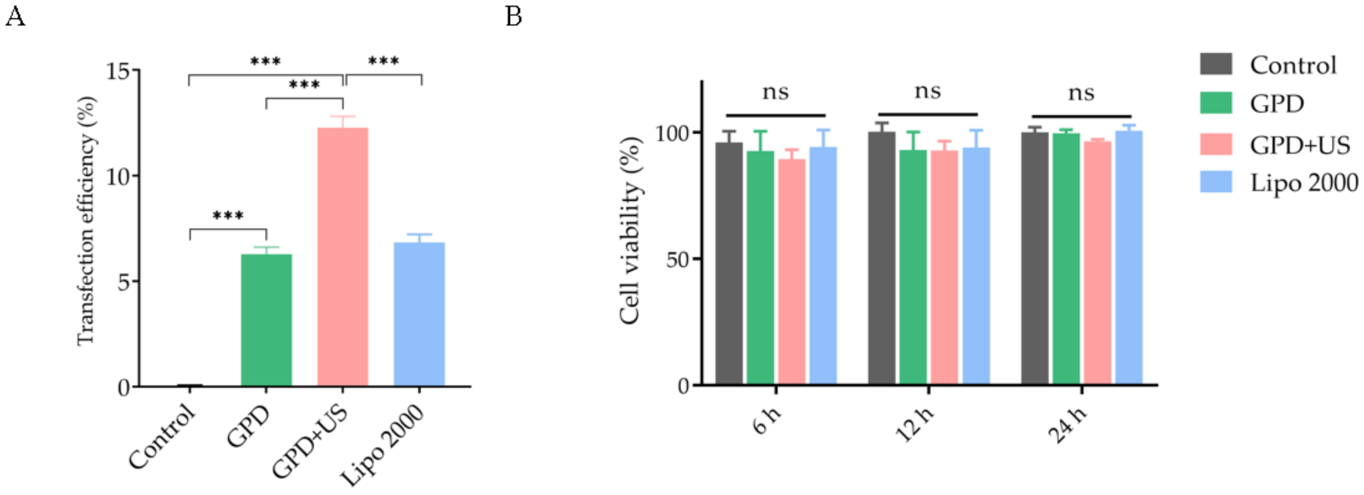

Figure S2. Transfection efficiency and cytotoxicity of GVs–PEI–DNA(GPD) vs lipofectamine 2000. (A) Quantitative fluorescence analysis of Cas9- and EGFP-stably expressed 4T1 cells transfected with only pU6-sgRNA(Cdh2)-mCherry(control), GPD, GPD + US, or lipofectamine 2000. (n = 3). (B) Cell viability of Cas9- and EGFP-stably expressed 4T1s determined through the CCK-8 assay. (n = 3). ns denotes P > 0.05, ***P < 0.001.

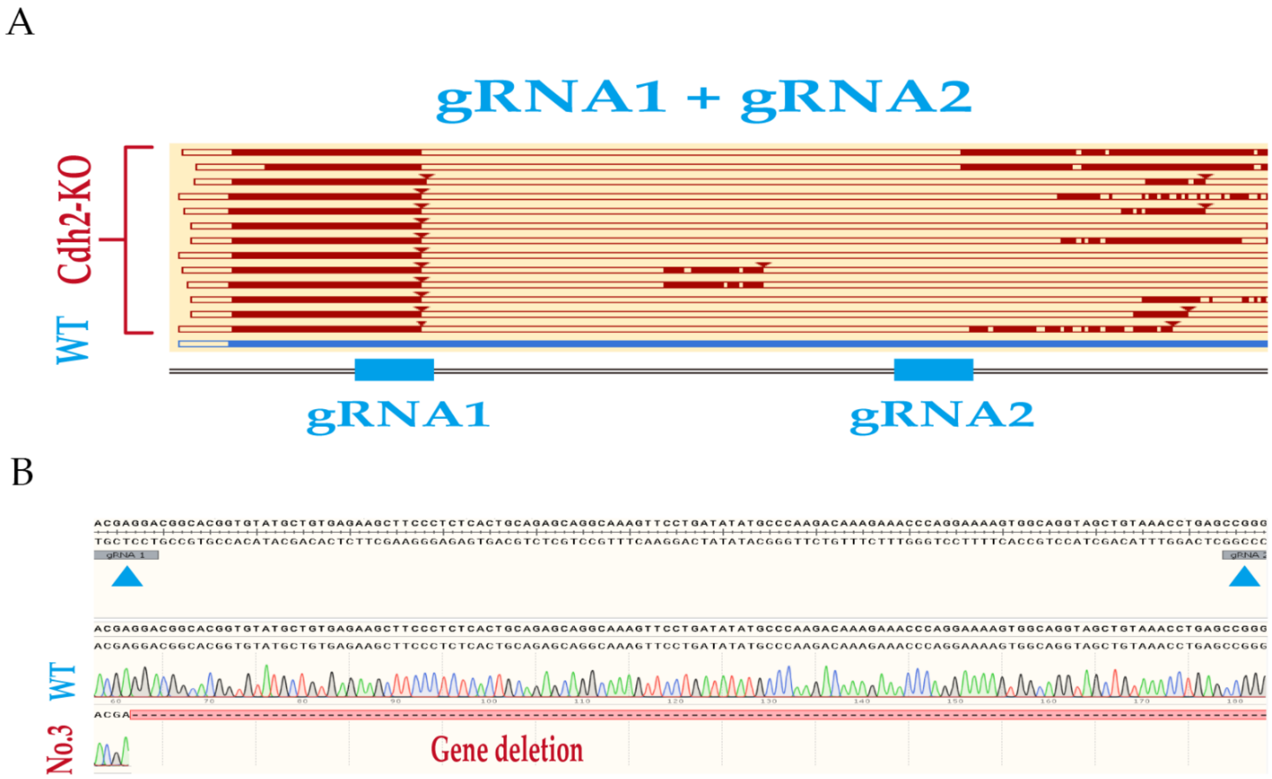

Figure S3. Sanger sequencing results of wild-type (WT) and Cdh2-edited cell lines. (A) Sanger sequencing results of 13 cell lines with gene mutations in target regions. gRNA1 and gRNA2 were designed as two target sites. (B) Sequencing peaks for WT and one of the Cdh2-edited cell lines, No.3.
